# Efficacy and safety of cannabidiol for the treatment of canine osteoarthritis: a systematic review and meta-analysis of animal intervention studies

**DOI:** 10.3389/fvets.2023.1248417

**Published:** 2023-09-15

**Authors:** Chanthawat Patikorn, Osot Nerapusee, Kumpanart Soontornvipart, Kanta Lawonyawut, Kachapong Musikpodok, Kanisorn Waleethanaphan, Puree Anantachoti

**Affiliations:** ^1^Department of Social and Administrative Pharmacy, Faculty of Pharmaceutical Sciences, Chulalongkorn University, Bangkok, Thailand; ^2^Department of Veterinary Surgery, Faculty of Veterinary Science, Chulalongkorn University, Bangkok, Thailand

**Keywords:** *Cannabis sativa*, cannabidiol, canine osteoarthritis, dogs, pain

## Abstract

**Introduction:**

Canine osteoarthritis (OA) is a degenerative disease with chronic inflammation of internal and external joint structures in dogs. *Cannabis* spp. contains cannabidiol (CBD), a substance known for various potential indications, such as pain relief and anti-inflammatory in various types of animals, including dogs with OA. As CBD is increasingly in the spotlight for medical use, we aimed to perform a systematic review and meta-analysis to evaluate the efficacy and safety of CBD in treating canine OA.

**Methods:**

We searched PubMed, Embase, Scopus, and CAB Direct for animal intervention studies investigating the effects of CBD for canine OA from database inception until February 28, 2023. Study characteristics and findings were summarized. A risk of bias in the included studies was assessed. Meta-analyses were performed using a random-effects model to estimate the effects of CBD on pain scores (0–10), expressed as mean difference (MD) and 95% confidence interval (95% CI). Certainty of evidence was assessed using GRADE.

**Results:**

Five articles were included, which investigated the effects of CBD in 117 dogs with OA. All studies were rated as having a high risk of bias. CBD products varied substantially, i.e., oral full-spectrum CBD oil in four studies, and isolated CBD oil and liposomal CBD oil in another study. Treatment duration varied from 4–12 weeks. Meta-analyses of three studies found that, in dogs with OA, treatment with oral full-spectrum CBD oil may reduce pain severity scores (MD; −0.60, 95% CI; −1.51 to 0.31, *I*^2^ = 45.64%, *p* = 0.19) and pain interference scores (MD; −1.52, 95% CI; −3.84 to 0.80, *I*^2^ = 89.59%, *p* = 0.20) but the certainty of evidence was very low. CBD is generally considered safe and well-tolerated in the short-run, with few mild adverse events observed, such as vomiting and asymptomatic increase in alkaline phosphatase level.

**Conclusion:**

CBD is considered safe for treating canine OA. CBD may reduce pain scores, but the evidence is very uncertain to conclude its clinical efficacy. High-quality clinical trials are needed to further evaluate the roles of CBD in canine OA.

## 1. Introduction

Canine osteoarthritis (OA) is a degenerative disease of the joint in dogs expressed by chronic inflammation of internal and external joint structures. Pain, swelling, and joint deformity are usually observed among OA patients, which limits movement and eventually leads to irreversible disability (1). There are five stages (0–4) of canine OA according to the COAST system, including stage 0—preclinical dogs without apparent risk factors for OA, stage 1—preclinical dogs with risk factors for OA, stage 2—mild OA dogs, stage 3—moderate OA dogs, and stage 4—severe OA dogs (2). The prevalence of canine OA was reported to be up to 20% in the UK and the US. Canine OA is one of the leading chronic diseases affecting dogs’ quality of life (3, 4).

The cause of OA in dogs is still unknown. Risk factors associated with disease progression can be classified into two categories: non-modifiable risk factors, such as age and large breeding, and modifiable risk factors, such as weight (1). OA is chronic and cannot be cured. The treatment goal is to slow the disease progression, relieve pain, and improve the dog’s quality of life (4).

Pain management can often be divided into two stages: acute and chronic stages. Mild to moderate pain in the acute OA stage can be well managed by analgesic drugs, whereas moderate to severe pain is managed by multimodal analgesia, which includes opioids, nonsteroidal anti-inflammatory drugs (NSAIDs), local anesthetics, N-methyl-D-aspartate (NMDA) receptor antagonists, and alpha 2-adrenoceptor agonists. Short-term applications of NSAIDs are regarded as the mainstay of treatment for chronic stages, along with non-pharmacological interventions such as acupuncture or surgery (5). However, NSAIDs are not effective for chronic pain and for long-term use. They increase the risk of peptic ulcer and renal failure, especially among the elderly and those with kidney and gastrointestinal diseases (6).

Dietary supplements using omega-3 fatty acids may aid in managing canine OA in the chronic phase. Studies showed that compared to the control group, dogs receiving omega-3 fatty acids had a significant decrease in carprofen dosage (7). Moreover, a recent systematic review and meta-analysis showed an evident clinical analgesic efficacy of omega-3 fatty acids in canine OA (8).

*Cannabis* spp. was previously strictly controlled because it contains delta-9-tetrahydrocannabinol (THC), a psychoactive compound (9). Because of THC’s addictive properties, previous clinical studies were quite limited. Besides THC, another key active ingredient in *Cannabis* spp. is cannabidiol (CBD). CBD is known for various potential indications such as pain relief, anti-inflammatory, anti-epilepsy, anti-neoplastic, and anti-depression properties (10–13). Because of the positive benefits of the CBD, the United States Department of Agriculture (USDA) enacted the 2018 Farm Bill. This legislation removed hemp, defined as *Cannabis sativa* L., with a low THC content (less than 3% THC on a dry weight basis) from the list of controlled substances (14). As a result, hemp products and active compounds such as CBD extracts have emerged in both the human and animal healthcare markets.

Many studies investigate the pharmacological activity of CBD in various types of animals, including dogs. CBD is an allosteric non-competitive antagonist with an affinity to bind with cannabinoid receptors. These cannabinoid receptors are found in many locations of dogs, such as the central nervous system, peripheral nervous system, cardiovascular system, immune system, gastrointestinal system, reproductive system, skin, and synovial fluid (15–17). As a result, CBD could be used as a new alternative therapy to relieve pain in dogs suffering from OA. A pharmacokinetic study of CBD in dogs with OA revealed that the half-life (T_1/2_) of CBD were approximately 4 h for both CBD 2 mg/kg and 8 mg/kg, which were given every 12 h (18). Many factors influence CBD bioavailability and metabolism, including product formulations, dosage forms, routes of administration, and whether they are administered fast or with food. It was found that co-administration of CBD with food might increase CBD absorption (19, 20). Individualized patients’ breed, age, fat percentage, and body condition all play a role in CBD pharmacokinetics (18, 21). However, the results of pharmacokinetic studies alone were inadequate to support clinical use.

At present, only a limited number of studies have evaluated the efficacy and safety of CBD in dogs with OA. These prior studies unanimously reported a few adverse events, which were not serious ones (18, 22–27). The literature contains several review articles; however, those previous reviews did not perform meta-analysis (9, 28, 29). Hence, this study aimed to perform a systematic review and meta-analysis of available clinical trials to investigate the efficacy and safety of CBD in the treatment of canine OA, as CBD is increasingly in the spotlight for medical use.

## 2. Materials and methods

The protocol of this systematic review was registered with PROSPERO (CRD42023381113) (30). This study was conducted following the Cochrane handbook for systematic reviews of interventions (31) and reported following the 2020 preferred reporting items for systematic reviews and meta-analyses (PRISMA) reporting guideline as shown in Supplementary Table S1 (32).

### 2.1. Eligibility criteria

Animal intervention studies that met the eligibility criteria described in the population, intervention, control, outcome (PICO) framework were included. *Population*: dogs with OA regardless of breed, age, weight, comorbidity, and pre-existing treatment. *Intervention*: any forms (route of administration, dose, and duration) of CBD. CBD products were categorized based on the molecular content, including (1) full-spectrum; a product that contains CBD and all-natural compounds found in the *Cannabis* plant, (2) broad-spectrum; a product that contains CBD and all-natural compounds except for tetrahydrocannabinol (THC), and (3) isolated; a product contains only natural CBD compound. *Comparators*: any comparators. *Outcomes*: any efficacy outcomes of the articles, such as pain measurement, positions, and movement, and safety outcomes, such as adverse events. We excluded articles that have no full-text article available.

### 2.2. Search strategy

We searched PubMed, Embase, Scopus, and CAB Direct for studies relevant to eligibility criteria from database inception until February 28, 2023. Our search terms were; (*Cannabis* OR hemp OR hempseed OR hemp seed OR *Cannabis* OR marijuana OR *Cannabis sativa* OR cannabinoids OR delta-9-tetrahydrocannabinol OR cannabidiol OR cannabinol OR weed OR CBD OR THC) AND (dogs OR dog OR canine OR canines) AND (arthritis OR osteoarthritis OR OA), that was adapted to match the search techniques of each database. A full search strategy is shown in Supplementary Table S2. For this systematic review, there were no limitations on language. Additionally, we conducted a supplementary search by tracking citations and examining the reference lists of relevant articles.

### 2.3. Study selection

Articles retrieved using the mentioned search strategy were imported to EndNote version 20. Duplicates were removed. Two reviewers independently assessed the titles and abstracts of the studies to see if they met the inclusion criteria. We then retrieved the full text of potentially eligible studies and had two reviewers independently assess their eligibility. If the reviewers disagreed on a study’s eligibility, a third reviewer was consulted to resolve the issue.

### 2.4. Data extraction

A standardized, pre-piloted form was used to extract data from the included studies. Two reviewers independently extracted data from each article. The following data were extracted; first author, publication year, study design, study duration, participant characteristics, dosage form, strength, dosing regimen, route of administration, and components of CBD products, efficacy measures, and adverse events.

### 2.5. Quality assessment

Randomized controlled trials (RCTs) were assessed for risk of bias using a risk of bias tool for animal intervention studies (SYRCLE’s RoB tool) (33), which addressed six types of bias: (1) selection bias; (2) performance bias; (3) detection bias; (4) attrition bias; (5) reporting bias; and (6) other, across 10 domains. Each domain was categorized as “high,” “unclear,” or “low” risk of bias.

Non-randomized studies were evaluated by the methodological index for non-randomized studies (MINORS) (34), which defined eight specific domains: (1) clearly stated aim; (2) inclusion of consecutive patients; (3) prospective data collection; (4) endpoints appropriate to study aim; (5) unbiased assessment of study endpoint; (6) follow-up period appropriate to study aim; (7) <5% lost to follow-up; (8) prospective calculation of study size.

### 2.6. Data analysis

A narrative descriptive analysis was performed to summarize the characteristics of the included studies. We performed meta-analyses using a random-effects model under Dersimonian and Laird method to estimate the effects of CBD on efficacy measures (35). Mean and standard deviation (SD) at the last visit of treatment and control groups of the included studies were used to calculate the pooled effects of CBD. Effect sizes were reported as mean difference (MD) and its 95% confidence interval (95% CI) and presented in a forest plot. Type-I error was fixed at 5% and two-sided.

We tested for heterogeneity assessed by *χ*^2^ and *I*^2^ statistic index. The *I*^2^ value ranges between 0% and 100% and is typically determined low when <40%, modest when 30%–60%, substantial when 50%–90%, and considerable when 75%–100% (31). Sensitivity meta-analyses were performed by removing studies assessed as high risk of bias to evaluate the robustness of the findings. Publication bias was assessed using the Egger regression asymmetry test (36). Statistical analyses were performed using STATA version 18.0.

### 2.7. Certainty assessment

We assessed the certainty of the evidence (CoE) of the meta-analyzed effects of CBD on pain outcomes. The CoE was assessed using the grading of recommendations, assessment, development and evaluation (GRADE) approach, which included the assessment of the risk of bias, inconsistency, indirectness, imprecision, and publication bias. GRADE has four levels of CoE, including very low, low, moderate, and high (37).

## 3. Results

### 3.1. Study selection

**Figure 1:**
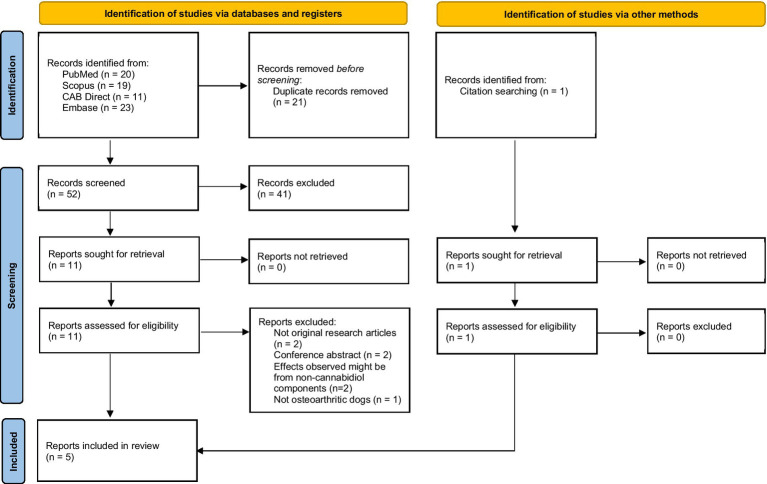
Study selection flow diagram (32).

A total of 73 articles were identified from the search. Five studies investigating the efficacy and safety of CBD products were included (18, 22, 24, 26, 27). The study selection flow is presented in Figure 1. A list of excluded studies with reasons is presented in Supplementary Table S3. We exclude a clinical trial investigating oral tablet containing full-spectrum CBD oil and *Boswellia serrata* Roxb. and *Zingiber officinale* phytosomized because the effects observed might not be from CBD (23, 25).

### 3.2. Study characteristics

Table 1 summarizes the characteristics of the included studies, which involved 117 dogs with OA. Two studies were parallel RCTs (22, 27), while the other two were cross-over RCTs (18, 26). One study was a single-arm study (24). Participating dogs were client-owned dogs with OA, of which three studies specifically included only dogs with radiographic-confirmed OA (18, 22, 26). Affected joints were reported in 63 dogs in three studies (18, 22, 26), of which the most commonly affected joints were the elbow (57%), hip (32%), stifle (27%), shoulder (8%), antebrachiocarpal (2%), and coxofemeral (2%).

**Table 1:** Characteristics of included studies.

First author, year	Study design	Study population	Intervention (*n*)	Control (*n*)	Treatment duration	Funding source
**Single arm study**						
Kogan, 2020 (24)	Single arm	Client-owned dogs with osteoarthritis	Oral full-spectrum CBD oil 0.25 mg/kg once daily for 3 days, then escalated to 0.50–0.75 mg/kg every 12 h until pain severity score (0–10) was 0–1 (*n* = 37) (dogs were allowed to continue using gabapentin, polysulfated aminoglycan, and acupuncture)	No control group	12 weeks	Not reported
**Parallel randomized controlled study**						
Brioschi, 2020 (22)	- Parallel - Randomized - Owner blinded	Client-owned dogs with radiographic-confirmed osteoarthritis	Oral transmucosal full-spectrum CBD oil 2 mg/kg every 12 h plus standard of care [combination of NSAIDs (or corticosteroid if NSAIDs were contraindicated), gabapentin, and amitriptyline] (*n* = 9)	Standard of care [combination of NSAIDs (or corticosteroid if NSAIDs were contraindicated), gabapentin, and amitriptyline] (*n* = 12)	12 weeks	No external funding received
Verrico, 2020 (27)	- Parallel - Randomized - Placebo-controlled - Veterinarian and owner blinded	Client-owned dogs with osteoarthritis	- Oral isolated CBD oil 0.5 mg/kg every 12 h (*n* = 5) - Oral isolated CBD oil 1.2 mg/kg every 12 h (*n* = 5) - Oral liposomal CBD oil 0.5 mg/kg every 12 h (*n* = 5)	Placebo oil (fractionated coconut oil) (*n* = 5)	4 weeks	-Medterra CBD, Inc. - Baylor College of Medicine—NIH
**Cross-over randomized controlled study**						
Gamble, 2018 (18)	- Cross-over - Randomized - Placebo-controlled - Veterinarian and owner blinded	Client-owned dogs with radiographic-confirmed osteoarthritis	Oral full-spectrum CBD oil 2 mg/kg every 12 h plus standard of care (NSAIDs, fish oil, and/or glucosamine sulfate, chondroitin sulfate) (*n* = 16)	Placebo oil (olive oil with 10 PPT anise oil and 5 PPT peppermint oil) plus standard of care (NSAIDs, fish oil, and/or glucosamine sulfate, chondroitin sulfate) (*n* = 16)	10 weeks (4-2-4)^*^	Ellevet LLC
Mejia, 2021 (26)	- Cross-over - Randomized - Placebo-controlled - Veterinarian and owner blinded	Client-owned dogs with radiographic-confirmed osteoarthritis	Oral full-spectrum CBD oil 2.5 mg/kg every 12 h (*n* = 23)	Placebo oil (*n* = 23)	12 weeks (6-0-6)^*^	Not reported

^*^Three numbers in parenthesis indicate (1) duration of treatment A, (2) wash-out period before cross-over, and (3) duration of treatment B. CBD, cannabidiol; NSAIDs, nonsteroidal anti-inflammatory drugs; PPT, parts per thousands.

Dog breed was reported in five studies (18, 22, 24, 26, 27), with the most common dog breed being large dog breeds, such as Labrador Retriever (*n* = 19), German shepherd (*n* = 8), and Australian shepherd (*n* = 5). The average weight of dogs reported in four articles was 28.14 kg (SD 11.99) (18, 22, 24, 27). The average age of dogs reported in five studies was 10.9 years (SD 2.86) (18, 22, 24, 26, 27).

CBD products investigated in the included studies varied substantially, i.e., full-spectrum CBD oil (*n* = 4) (18, 22, 24, 26), isolated CBD oil (*n* = 1) (27), and liposomal CBD oil (*n* = 1) (27). The route of administration was oral (*n* = 4) (18, 24, 26, 27), or oral transmucosal (*n* = 1) (22). CBD products were either used alone (*n* = 2) (26, 27) or as part of a multimodal treatment (*n* = 3) (18, 22, 24).

The commonly used dose of CBD was 2–2.5 mg/kg every 12 h (*n* = 3) (18, 22, 26). One study investigated the effects of oral full-spectrum CBD oil 0.25 mg/kg once daily with dose escalation to 0.50–0.75 mg/kg every 12 h (24). Treatment duration varied from 4–12 weeks.

### 3.3. Quality assessment

Quality assessment is presented in Supplementary Table S4. Overall, all five studies were considered as having a high risk of bias, as at least one domain was rated as having a high risk of bias. Cross-over RCTs were primarily rated as having a high risk of bias arising from the washout period and carryover effects, and unequal distribution of baseline characteristics (18, 26). Parallel RCTs were primarily rated as having a high risk of bias due to the selection of the reported result, unequal distribution of baseline, and lack of blinding (22, 27). The single-arm study was rated as having a high risk of bias (11/12 points) due to a biased assessment of the study endpoint and unreported prospective calculation of the study size (24).

### 3.4. Efficacy and safety of CBD products

We provided a summary of the efficacy and safety findings of CBD products in Supplementary Table S5. Overall outcome measures of the included studies were grouped into four domains: (1) pain, (2) activity and locomotion, (3) quality of life, and (4) safety.

#### 3.4.1. Pain

Various pain assessment tools were selected across the studies; pain severity score (*n* = 1) (24), canine brief pain inventory (CBPI) (*n* = 3) (18, 22, 26), Liverpool osteoarthritis in dogs (LOAD) (*n* = 1) (26), and pain scores assessed by veterinarians (*n* = 1) (18).

**Figure 2:**
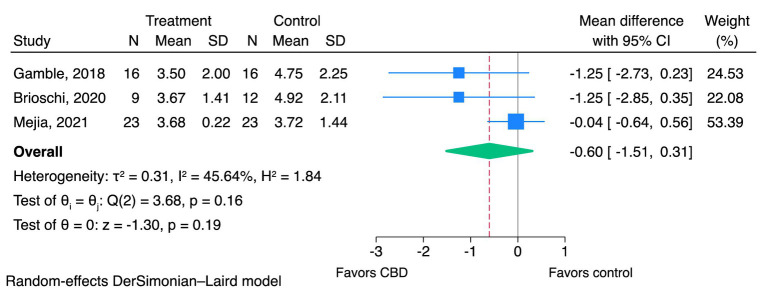
Effects of CBD on pain severity using CBPI—PSS (0–10). CBD, cannabidiol; CBPI, canine brief pain inventory; PSS, pain severity score.

**Figure 3:**
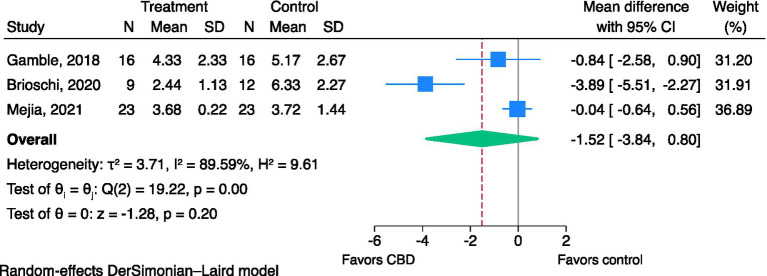
Effects of CBD on pain interference using CBPI—PIS (0–10). CBD, cannabidiol; CBPI, canine brief pain inventory; PIS, pain interference score.

Meta-analyses were performed to pool the effect sizes of CBD products on pain scores from the same measurement reported in two or more studies. The analysis was only possible for CBPI pain severity score (PSS) and CBPI pain interference score (PIS) reported in three studies (18, 22, 26), as shown in Figures 2, 3. The results of the assessment of the CoE are shown in Table 2. CBPI contained 11 domains that questioned dog owners to assess their dogs in the last 7 days. CBPI can be divided into three main groups of the question; pain severity (PSS; 0–10): four domains to assess the pet’s pain, pain interference with function (PIS; 0–10): six domains to assess general activity, and quality of life (0–5): one domain to assess the dog’s overall quality of life (38). The reported CBPI PSS and CBPI PIS on a scale of 0–40 and 0–60 were converted into a scale of 0–10 by dividing the scores by 4 and 6, respectively.

**Table 2:** Grading the certainty of the evidence of the effects of cannabidiol on pain.

Certainty assessment							Summary of findings
Participants (studies) follow-up	Risk of bias	Inconsistency	Indirectness	Imprecision	Publication bias	Overall certainty of evidence	Mean difference (95% CI)
**Pain severity score (follow-up: range 6 weeks to 12 weeks; assessed with: CBPI—PSS; scale from: 0 to 10)**							
99 (3 RCTs)	Very serious^a^	Not serious	Not serious	Serious^b^	None	⨁ ◯◯◯ very low	−0.60 [−1.51, 0.31]
**Pain interference score (follow-up: range 6 weeks to 12 weeks; assessed with: CBPI—PIS; scale from: 0 to 10)**							
99 (3 RCTs)	Very serious^a^	Not serious	Not serious	Serious^b^	None	⨁ ◯◯◯ very low	−1.52 [−3.84, 0.80]

The certainty of the evidence was assessed using the grading of recommendations, assessment, development and evaluation (GRADE) approach. CI, confidence interval; CBPI, canine brief pain inventory; PIS, pain interference score; PSS, pain severity score.

a There was a high risk of bias from selecting the reported results and carryover effects.

b There was a relatively small number of samples, limiting the precision of the pooled estimates.

##### 3.4.1.1. Pain severity scores

Three studies were included in the meta-analysis (18, 22, 26). CBD may reduce pain severity scores, but the evidence is very uncertain (very low CoE; MD of CBPI PSS; −0.60, 95% CI; −1.51 to 0.31, *I*^2^ = 45.64%, *p* = 0.19, Figure 2). Egger’s test (*p* = 0.06) indicated no evidence of small-study effects. The *I*^2^ value indicated modest heterogeneity among the three studies. Sensitivity meta-analyses were not performed as all three studies were rated as having a high risk of bias.

The effects of CBD products on other pain severity scores were as follows. Gamble et al. revealed that dogs with OA in the full-spectrum CBD oil with the standard of care group did not show a significant improvement in the pain scores assessed by veterinarians, compared to the standard of care group (18). Kogan et al. (24) found that full-spectrum CBD oil significantly reduced pain severity scores by 2.23 units (SD 2.3) from baseline. Mejia et al. (26) found that dogs with OA in the full-spectrum CBD oil group compared to the placebo group did not show a statistically significant difference in terms of LOADs.

##### 3.4.1.2. Pain interference scores

Three studies were included in the meta-analysis (18, 22, 26). CBD may reduce pain interference scores, but the evidence is very uncertain (very low CoE: MD of CBPI PIS; −1.52, 95% CI; −3.84 to 0.80, *I*^2^ = 89.59%, *p* = 0.20, Figure 3). Egger’s test (*p* = 0.44) indicated no evidence of small-study effects. The *I*^2^ value indicated considerable heterogeneity. Sensitivity meta-analyses were not performed as all three studies were rated as having a high risk of bias.

#### 3.4.2. Activity and locomotion

Activity and locomotion were measured as total activity count and objective gait analysis (measured as % peak vertical force normalized by body weight and % body weight distribution) in one study (26), veterinarian-assessed lameness scores and weight-bearing scores in one study (18). However, no statistically significant change in activity and locomotion was observed between the CBD group and the control group in these studies.

#### 3.4.3. Quality of life

Quality of life was reported in one study, which found that dogs with OA in the oral transmucosal full-spectrum CBD oil plus standard of care group had higher quality of life index than the standard of care group. However, this difference was not statistically significant (22).

#### 3.4.4. Safety

##### 3.4.4.1. Laboratory parameters

There were no significant changes to the laboratory parameters, such as complete blood count, renal function test, or metabolic panel, between dogs with OA in the CBD group and the control group in four studies (18, 22, 27).

There was no change in liver enzymes observed in three studies (22, 27). However, significant elevation in liver enzymes was observed in three studies (18, 24, 26). Full-spectrum CBD oil significantly increased alkaline phosphatase (ALP) levels from 133.3 U/L at baseline to 264 U/L at week 12, which was higher than the normal values (24). Full-spectrum CBD oil plus standard of care group had higher ALP levels than the standard of care group at week 4 (323 U/L vs. 175 U/L) (18). Lastly, 14 dogs in the full-spectrum CBD oil group experienced an elevation in ALP levels compared to one dog in the placebo group (61% vs. 4%). Among the 14 dogs, six had increased alanine aminotransferase (ALT) levels, and three had increased aspartate aminotransferase (AST) levels. None of these dogs displayed clinical symptoms, and concomitant treatment was continued without further owner-reported adverse events (26).

##### 3.4.4.2. Adverse events

Adverse events were reported in three studies (22, 26). CBD products were generally well tolerated, with mild or absent gastrointestinal side effects reported. Brioschi et al. (22) reported dogs experiencing minimal ptyalism (2/9, 22%), somnolence and mild ataxia (1/9, 11%), from receiving oral transmucosal full-spectrum CBD oil plus standard of care (22). Mild vomiting (1/24, 4%) and vomiting leading to treatment discontinuation (1/24, 4%) were reported in dogs receiving full-spectrum CBD oil (26).

## 4. Discussion

CBD, a non-psychotropic compound found in the *Cannabis sativa* L. plant, has been studied with the hope of finding an alternative treatment for canine OA. We performed a systematic review and meta-analysis to evaluate the efficacy and safety of CBD products in dogs with OA. A total of five studies were identified, which evaluated a wide range of CBD products, including oral full-spectrum CBD oil, oral transmucosal full-spectrum CBD oil, isolated CBD oil, and liposomal CBD oil. The duration of treatment for these products ranged from 4–12 weeks.

CBD products can differ greatly. The CBD products used in the study varied from the full-spectrum CBD formulations, which contain other active substances with synergistic effect (39). This is in contrast to isolated CBD formulations that only contain CBD (18). Moreover, the different routes of CBD administration may affect its bioavailability and absorption. Compared with the oral route, the oral transmucosal route may reduce first-pass metabolism of CBD and raise CBD plasma levels in participating dogs which increases its anti-inflammatory effect on injured joints and is associated with improvements in the pain control (22).

The meta-analyses of three studies found that CBD may reduce pain scores, but the evidence is very uncertain. There was a high risk of bias from selecting the reported results and carryover effects and a relatively small number of samples, limiting the precision of the pooled estimates. These indicated the need for high-quality clinical trials to further investigate the role of CBD on pain control in dogs with OA.

According to the treatment of canine OA in clinical practice, it needs multimodal treatment, including physiotherapy, NSAIDs (for acute pain in chronic OA patients), gabapentin/amitriptyline (for allodynia treatment and as adjuvant treatment for pain), and nutraceuticals (omega-3 fatty acids) (5). In some studies, dogs were allowed to be treated with multimodal therapy (18, 22, 24). These co-interventions may impact the treatment outcomes. As shown in the meta-analyses in Figures 2, 3, studies of CBD with a multimodal treatment (18, 22) showed a trend that there was a relatively higher magnitude of the reduction of pain scores than a study of CBD oil monotherapy (26). The concomitant administration of CBD and NSAIDs may result in a prolonged duration of the effects of CBD. This is due to the involvement of cyclooxygenase type-2 (COX-2) in the metabolism of CBD. Therefore, NSAIDs that selectively inhibit COX-2 may impede the breakdown of CBD, leading to a longer duration of its pharmacological activity (40). Nonetheless, to the best of our knowledge, there was no study in dogs investigating the interaction between CBD and gabapentin, amitriptyline, fish oil, glucosamine sulfate, and chondroitin sulfate.

CBD is generally considered safe and well-tolerated, with few mild adverse events observed such as vomiting. Although the ALP levels had abnormally risen, the clinical adverse symptoms related to that abnormal value were not observed. Furthermore, ALT levels remained unchanged, supporting the lack of hepatocellular damage. Therefore, hepatotoxic potential of CBD cannot be determined (18, 24, 26). Increased ALP levels might be associated with the induction of cytochrome P-450 mediated oxidative metabolism of the liver as a result from receiving *Cannabis* spp. extract (41, 42).

Due to increase in ALP levels without increased ALT, other associated causes, such as cholestasis or a progression of regenerative nodular hyperplasia of the live cannot be ruled out (18). On the other hand, concurrent use of NSAIDs also might play a role in the ALP elevation. Eight of thirteen dogs receiving concomitant NSAIDs therapy throughout the study period had significantly risen in ALP levels during CBD administration (26). Even though the interaction between CBD and NSAIDs has not been studied in dogs, NSAIDs were found to be associated with liver enzyme elevation in dogs (6). Future studies should focus on liver function enzymes over a longer period.

The findings of this systematic review and meta-analysis should be interpreted under the following limitations. There was a relatively small number of participating dogs in the clinical studies which might not be enough to fully establish the clinical efficacy of CBD in treating canine OA. There was heterogeneity in terms of the different research designs across the included studies. Different study designs (cross-over, parallel, single arm), no run-in before starting the trial, varying washout period in cross-over study (no washout period and 2 weeks), using different strengths of CBD (less than 1 mg/kg, 2 mg/kg, and 2.5 mg/kg), using different dosage form (oral oil, transmucosal, and tablet), allow co-existing treatment (NSAIDs, corticosteroid, gabapentin, amitriptyline, fish oil, polysulfated glycosaminoglycan, glucosamine sulfate, chondroitin sulfate, and acupuncture), length of study (from 4–12 weeks) and using a different approach to quantify the primary outcome (subjective report of pain scores by dog owners, objective gait analysis, using different pain measurement) were observed among five included studies. Moreover, all the included studies were rated as having a high risk of bias, thus affecting the credibility of the findings of individual studies.

Findings from this study may encourage potential clinical use and endorse the commercialization of CBD in the future. However, further research with appropriate design is needed to better provide stronger evidence of the efficacy and safety of CBD and facilitate conducting an updated meta-analysis in the future. Therefore, we proposed some features to be considered in the clinical studies of CBD products. Firstly, a parallel trial is preferred over a crossover trial to avoid the carryover effect. Because there were still no long-term steady-state pharmacokinetics studies in dogs to determine an appropriate washout period to ensure the elimination of CBD, conducting the crossover trial may pose a higher risk of carryover effect than the parallel trial. Secondly, attempts should be made to mask the unique smell of CBD to ensure treatment blinding to avoid ascertainment bias. Lastly, objective measures, such as force plate gait analysis (43) and infrared thermal imaging (44), are preferred over subjective measures to demonstrate the clinical efficacy of CBD.

## 5. Conclusion

CBD is considered safe for treating dogs with OA in the short run. CBD may reduce pain scores, but the evidence is very uncertain to conclude its clinical efficacy. High-quality randomized controlled trials are needed to further evaluate the roles of CBD in treating canine OA, especially the long-term efficacy. Pharmacovigilance is recommended after the initial product launch to monitor the safety profile of CBD products.

## Data availability statement

The original contributions presented in the study are included in the article/Supplementary material, further inquiries can be directed to the corresponding author.

## Author contributions

CP and PA conceptualized the study. KL, KM, and KW developed the review protocol and performed the systematic review under CP, ON, KS, and PA supervision. CP performed statistical analyses. CP, KL, KM, and KW wrote the first draft of the manuscript. PA, ON, and KS reviewed, revised, and approved the publication of the manuscript. All authors contributed to the article and approved the submitted version.

## Funding

This study is funded by the Ratchadapiseksompotch Fund Chulalongkorn University, ReinUni_65_01_33_29. The funders had no role in study design, data collection, data analysis, data interpretation, writing of the report, or the decision to submit for publication.

## Conflict of interest

The authors declare that the research was conducted in the absence of any commercial or financial relationships that could be construed as a potential conflict of interest.

## Publisher’s note

All claims expressed in this article are solely those of the authors and do not necessarily represent those of their affiliated organizations, or those of the publisher, the editors and the reviewers. Any product that may be evaluated in this article, or claim that may be made by its manufacturer, is not guaranteed or endorsed by the publisher.
