# Supplementary material for: Efficacy and safety of cannabidiol for the treatment of canine osteoarthritis: a systematic review and meta-analysis of animal intervention studies
Source: Front Vet Sci. 2023 Sep 15;10:1248417. doi: 10.3389/fvets.2023.1248417 (PMC10540436; doi:10.3389/fvets.2023.1248417)
Supplement: Supplementary file 1 [file Table_1.DOCX]

**Efficacy and safety of cannabidiol for the treatment of canine osteoarthritis: a systematic review and meta-analysis of animal intervention studies**

**SUPPLEMENTARY MATERIAL**

**Table S1. PRISMA 2020 Checklist**

| **Section and Topic** | **Item #** | **Checklist item** | **Location where item is reported** |
| --- | --- | --- | --- |
| **TITLE** | | |  |
| Title | 1 | Identify the report as a systematic review. | Title |
| **ABSTRACT** | | |  |
| Abstract | 2 | See the PRISMA 2020 for Abstracts checklist. | Abstract |
| **INTRODUCTION** | | |  |
| Rationale | 3 | Describe the rationale for the review in the context of existing knowledge. | Introduction |
| Objectives | 4 | Provide an explicit statement of the objective(s) or question(s) the review addresses. | Introduction |
| **METHODS** | | |  |
| Eligibility criteria | 5 | Specify the inclusion and exclusion criteria for the review and how studies were grouped for the syntheses. | Materials and Methods; Eligibility criteria |
| Information sources | 6 | Specify all databases, registers, websites, organisations, reference lists and other sources searched or consulted to identify studies. Specify the date when each source was last searched or consulted. | Materials and Methods; Search strategy |
| Search strategy | 7 | Present the full search strategies for all databases, registers and websites, including any filters and limits used. | SUPPLEMENTARY MATERIAL Table S2 |
| Selection process | 8 | Specify the methods used to decide whether a study met the inclusion criteria of the review, including how many reviewers screened each record and each report retrieved, whether they worked independently, and if applicable, details of automation tools used in the process. | Materials and Methods; Study selection |
| Data collection process | 9 | Specify the methods used to collect data from reports, including how many reviewers collected data from each report, whether they worked independently, any processes for obtaining or confirming data from study investigators, and if applicable, details of automation tools used in the process. | Materials and Methods; Data extraction |
| Data items | 10a | List and define all outcomes for which data were sought. Specify whether all results that were compatible with each outcome domain in each study were sought (e.g. for all measures, time points, analyses), and if not, the methods used to decide which results to collect. | Materials and Methods; Data extraction |
|  | 10b | List and define all other variables for which data were sought (e.g. participant and intervention characteristics, funding sources). Describe any assumptions made about any missing or unclear information. | Not Applicable |
| Study risk of bias assessment | 11 | Specify the methods used to assess risk of bias in the included studies, including details of the tool(s) used, how many reviewers assessed each study and whether they worked independently, and if applicable, details of automation tools used in the process. | Materials and Methods; Quality assessment |
| Effect measures | 12 | Specify for each outcome the effect measure(s) (e.g. risk ratio, mean difference) used in the synthesis or presentation of results. | Materials and Methods; Data analysis |
| Synthesis methods | 13a | Describe the processes used to decide which studies were eligible for each synthesis (e.g. tabulating the study intervention characteristics and comparing against the planned groups for each synthesis (item #5)). | Materials and Methods; Data analysis |
|  | 13b | Describe any methods required to prepare the data for presentation or synthesis, such as handling of missing summary statistics, or data conversions. | Materials and Methods; Data analysis |
|  | 13c | Describe any methods used to tabulate or visually display results of individual studies and syntheses. | Materials and Methods; Data analysis |
|  | 13d | Describe any methods used to synthesize results and provide a rationale for the choice(s). If meta-analysis was performed, describe the model(s), method(s) to identify the presence and extent of statistical heterogeneity, and software package(s) used. | Materials and Methods; Data analysis |
|  | 13e | Describe any methods used to explore possible causes of heterogeneity among study results (e.g. subgroup analysis, meta-regression). | Materials and Methods; Data analysis |
|  | 13f | Describe any sensitivity analyses conducted to assess robustness of the synthesized results. | Materials and Methods; Data analysis |
| Reporting bias assessment | 14 | Describe any methods used to assess risk of bias due to missing results in a synthesis (arising from reporting biases). | Materials and Methods; Data analysis |
| Certainty assessment | 15 | Describe any methods used to assess certainty (or confidence) in the body of evidence for an outcome. | Materials and Methods; Certainty assessment |
| **RESULTS** | | |  |
| Study selection | 16a | Describe the results of the search and selection process, from the number of records identified in the search to the number of studies included in the review, ideally using a flow diagram. | Results; study selection, Figure 1 |
|  | 16b | Cite studies that might appear to meet the inclusion criteria, but which were excluded, and explain why they were excluded. | SUPPLEMENTARY MATERIAL Table S3 |
| Study characteristics | 17 | Cite each included study and present its characteristics. | Results; study characteristics, Table 1, |
| Risk of bias in studies | 18 | Present assessments of risk of bias for each included study. | Results; Quality assessment, SUPPLEMENTARY MATERIAL Table S4 |
| Results of individual studies | 19 | For all outcomes, present, for each study: (a) summary statistics for each group (where appropriate) and (b) an effect estimate and its precision (e.g. confidence/credible interval), ideally using structured tables or plots. | SUPPLEMENTARY MATERIAL Table S5 |
| Results of syntheses | 20a | For each synthesis, briefly summarise the characteristics and risk of bias among contributing studies. | Results; study characteristics, quality assessment |
|  | 20b | Present results of all statistical syntheses conducted. If meta-analysis was done, present for each the summary estimate and its precision (e.g. confidence/credible interval) and measures of statistical heterogeneity. If comparing groups, describe the direction of the effect. | Results; efficacy and safety of CBD products, Figures 2, 3 |
|  | 20c | Present results of all investigations of possible causes of heterogeneity among study results. | Results, Pain |
|  | 20d | Present results of all sensitivity analyses conducted to assess the robustness of the synthesized results. | Results, Pain |
| Reporting biases | 21 | Present assessments of risk of bias due to missing results (arising from reporting biases) for each synthesis assessed. | Results, Pain |
| Certainty of evidence | 22 | Present assessments of certainty (or confidence) in the body of evidence for each outcome assessed. | Table 2. |
| **DISCUSSION** | | |  |
| Discussion | 23a | Provide a general interpretation of the results in the context of other evidence. | Discussion |
|  | 23b | Discuss any limitations of the evidence included in the review. | Discussion |
|  | 23c | Discuss any limitations of the review processes used. | Discussion |
|  | 23d | Discuss implications of the results for practice, policy, and future research. | Discussion |
| **OTHER INFORMATION** | | |  |
| Registration and protocol | 24a | Provide registration information for the review, including register name and registration number, or state that the review was not registered. | Materials and Methods |
|  | 24b | Indicate where the review protocol can be accessed, or state that a protocol was not prepared. | Materials and Methods |
|  | 24c | Describe and explain any amendments to information provided at registration or in the protocol. | Not Applicable |
| Support | 25 | Describe sources of financial or non-financial support for the review, and the role of the funders or sponsors in the review. | Funding |
| Competing interests | 26 | Declare any competing interests of review authors. | Conflict of interest |
| Availability of data, code and other materials | 27 | Report which of the following are publicly available and where they can be found: template data collection forms; data extracted from included studies; data used for all analyses; analytic code; any other materials used in the review. | Data availability statement |

From: Page MJ, McKenzie JE, Bossuyt PM, Boutron I, Hoffmann TC, Mulrow CD, et al. The PRISMA 2020 statement: an updated guideline for reporting systematic reviews. BMJ 2021;372:n71. doi: 10.1136/bmj.n71

**Table S2. Full search strategy**

| **Date** | **Database** | **Search term** | **Results** |
| --- | --- | --- | --- |
| 28 February 2023 | PubMed | (Cannabis OR hemp OR hempseed OR hemp seed OR cannabis OR marijuana OR cannabis Sativa OR cannabinoids OR delta-9-tetrahydrocannabinol OR cannabidiol OR cannabinol OR weed OR CBD OR THC) AND (dogs OR dog OR canine OR canines) AND (arthritis OR osteoarthritis OR OA) | 20 |
| 28 February 2023 | Scopus | ( TITLE-ABS-KEY ( cannabis OR hemp OR hempseed OR hemp AND seed OR cannabis OR marijuana OR cannabis AND sativa OR cannabinoids OR delta-9-tetrahydrocannabinol OR cannabidiol OR cannabinol OR weed OR cbd OR thc ) AND TITLE-ABS-KEY ( dogs OR dog OR canine OR canines ) AND TITLE-ABS-KEY ( arthritis OR osteoarthritis OR OA ) ) | 19 |
| 28 February 2023 | CAB Direct | (Cannabis OR hemp OR hempseed OR hemp seed OR cannabis OR marijuana OR cannabis Sativa OR cannabinoids OR delta-9-tetrahydrocannabinol OR cannabidiol OR cannabinol OR weed OR CBD OR THC) AND (dogs OR dog OR canine OR canines) | 11 |
| 28 February 2023 | Embase | (('hemp'/exp OR hemp OR 'hempseed'/exp OR hempseed OR 'hemp seed'/exp OR 'hemp seed' OR (('hemp'/exp OR hemp) AND ('seed'/exp OR seed)) OR 'cannabis'/exp OR cannabis OR 'marijuana'/exp OR marijuana OR 'cannabis sativa'/exp OR 'cannabis sativa' OR (('cannabis'/exp OR cannabis) AND sativa) OR 'cannabinoids'/exp OR cannabinoids OR 'delta 9 tetrahydrocannabinol'/exp OR 'delta 9 tetrahydrocannabinol' OR 'cannabidiol'/exp OR cannabidiol OR 'cannabinol'/exp OR cannabinol OR 'weed'/exp OR weed OR cbd OR thc) AND ('dogs'/exp OR dogs OR 'dog'/exp OR dog OR 'canine'/exp OR canine OR canines) AND ('arthritis'/exp OR arthritis OR 'osteoarthritis'/exp OR osteoarthritis OR oa)) AND [embase]/lim NOT ([embase]/lim AND [medline]/lim) | 23 |
|  |  | **Total** | **73** |

**Table S3. Excluded studies with reasons**

| **Reason for exclusion** | **Citation** |
| --- | --- |
| Not original research articles  (n = 2) | 1. Morrow L, Belshaw Z. Does the addition of cannabidiol alongside current drug treatments reduce pain in dogs with osteoarthritis? Vet Rec. 2020;186(15):493-4. 2. Morrow L, Belshaw Z. Is cannabidiol an effective supplementary treatment for reducing pain in dogs with osteoarthritis? Vet Rec. 2022;191(10):420-1. |
| Conference abstracts  (n = 2) | 1. Mejla S, Duerr FM, McGrath S. Evaluation of the effect of cannabidiol on osteoarthritis-associated pain in dogs-a pilot study. Veterinary and Comparative Orthopaedics and Traumatology. 2019;32. 2. Halpert M, Verrico C, Wesson S, Konduri V, Hofferek C, Vazquez-Perez J, et al. A randomized, double-blind, placebo-controlled study of daily cannabidiol for the treatment of canine osteoarthritis. Journal of Immunology. 2020;204(1). |
| Effects observed might be from non-cannabidiol components (n=2) | 1. Martello E, Biasibetti E, Bigliati M, et al. Preliminary results on the efficacy of a dietary supplement combined with physiotherapy in dogs with osteoarthritis on biomarkers of oxidative stress and inflammation. Ital J Anim Sci. 2021;20(1):2131-3. 2. Gabriele V, Bisanzio D, Riva A, et al. Long-term effects of a diet supplement containing Cannabis sativa oil and Boswellia serrata in dogs with osteoarthritis following physiotherapy treatments: a randomised, placebo-controlled and double-blind clinical trial. Nat Prod Res. 2022:1-5. |
| Not osteoarthritic dogs  (n = 1) | 1. Morris EM, Kitts-Morgan SE, Spangler DM, Gebert J, Vanzant ES, McLeod KR, et al. Feeding Cannabidiol (CBD)-Containing Treats Did Not Affect Canine Daily Voluntary Activity. Frontiers in Veterinary Science. 2021;8. |

**Table S4. Risk of bias assessment**

**A: Randomized controlled trials**

| **Type of bias** | **Domain** | **Cross-over trials** | | **Parallel trials** | |
| --- | --- | --- | --- | --- | --- |
|  |  | Gamble, 2018 | Mejia, 2021 | Verrico, 2020 | Brioschi, 2020 |
| Selection bias | Sequence generation | Low | Low | Low | Low |
|  | Baseline characteristics | High | High | High | Low |
|  | Allocation concealment | Unclear | Unclear | Unclear | Unclear |
| Performance bias | Random housing ^a^ | N/A | N/A | N/A | N/A |
|  | Blinding | Low | Low | Low | High |
| Detection bias | Random outcome assessment ^a^ | N/A | N/A | N/A | N/A |
|  | Blinding | Low | Low | Low | High |
| Attrition bias | Incomplete outcome data | Low | Low | Low | Low |
| Reporting bias | Selective outcome reporting | Low | High | High | High |
| Other | Other sources of bias | High ^b^ | High ^b^ | Low | Low |

a – these domains were not applicable as participating animals were client-owned dogs; b – bias arising from washout period and carryover effect.

**B: Single arm study**

| **Author, year** | | **Domain** | | | | | | | | |
| --- | --- | --- | --- | --- | --- | --- | --- | --- | --- | --- |
|  |  | **Domain 1:**  clearly stated aim | **Domain 2:**   inclusion of consecutive patients | **Domain 3:**   prospective collection of data | **Domain 4:**   endpoints appropriate to   the aim of the study | **Domain 5:**   unbiased assessment of   the study endpoint | **Domain 6:**   follow-up period   appropriate to the aim of   the study | **Domain 7:**   loss to follow up less than 5% | **Domain 8:**   prospective calculation of the   study size | **overall score** |
| Single arm study | Kogan, 2020 | 2 | 2 | 2 | 1 | 0 | 2 | 2 | 0 | 11 |

Note: The results of each following item were determined as scored 0 (not reported), 1 (reported but inadequate), or 2 (reported but adequate). The overall risk of bias was categorized as ‘high’ and ‘low’ risk bias when total scores of all domains were below 12 points and greater than or equal to 12 points, respectively.

**Table S5. Summary of findings on efficacy and safety outcomes**

| **First author, Year** | **Outcomes** | **Intervention** | **Baseline** | **Last visit** | **Control** | **Baseline** | **Last visit** |
| --- | --- | --- | --- | --- | --- | --- | --- |
| Kogan,  2020 | Pain severity score (0-10), mean ± SD | Oral full-spectrum CBD oil 0.25 mg/kg once daily for 3 days, then escalated to 0.50-0.75 mg/kg every 12 h until pain severity score (0-10) was 0-1  (n = 37) (dogs were allowed to continue using gabapentin, polysulfated aminoglycan, and acupuncture) | 3.2 ± 2.2 | 0.97 ± 0.81 | No control group | - | - |
|  | CBD dose (mg/kg), mean ± SD |  | 0.31 ± 0.04 | 1.67 ± 0.09 |  | - | - |
|  | Gabapentin dose (mg/day), mean ± SD |  | 1,846 ± 1,756 | 710 ± 1,112 |  | - | - |
|  | ALP (U/L), mean ± SD  (normal 17-111 U/L) |  | 133.3 ± 118 | 264 ± 233.2 |  | - | - |
|  | ALT (U/L), mean ± SD (normal 20-98 U/L) |  | 93.5 ± 69.3 | 91 ± 60.4 |  | - | - |
| Brioschi,  2020 | CBPI – PSS (0-10), mean ± SD | Oral transmucosal full-spectrum CBD oil 2 mg/kg every 12 h plus standard of care (combination of NSAIDs [or corticosteroid if NSAIDs were contraindicated], gabapentin, and amitriptyline)  (n = 9) | 5.33 ± 2.4 | 3.66 ± 1.4 | Standard of care (combination of NSAIDs [or corticosteroid if NSAIDs were contraindicated], gabapentin, and amitriptyline) (n = 12) | 5.83 ± 2.2 | 4.92 ± 2.1 |
|  | CBPI – PIS (0-10), mean ± SD |  | 6.33 ± 2.2 | 2.44 ± 1.1 |  | 7.25 ± 1.9 | 6.33 ± 2.3 |
|  | CBPI – QoL (0-4), mean ± SD |  | 2.55 ± 0.7 | 3.44 ± 0.7 |  | 2.25 ± 0.8 | 2.83 ± 0.9 |
|  | Ptyalism, n/N (%) |  | - | 2/9 (22%) |  | - | 0/12 (0%) |
|  | Somnolence and mild ataxia, n/N (%) |  | - | 1/9 (11%) |  | - | 2/12 (17%) |
|  | Blood cell count change, n/N (%) |  | - | 0/9 (0%) |  | - | 0/12 (0%) |
|  | Serum biochemistry change, n/N (%) |  | - | 0/9 (0%) |  | - | 0/12 (0%) |
| Verrico,  2020 | ALT (U/L), mean ± SD (normal 20-98 U/L) | Oral Isolated CBD 0.5 mg/kg every 12 h | 109.2 ± 78.7 | 85.2 ± 54.5 | Placebo oil (fractionated coconut oil) | 52.0 ± 19.7 | 63.25 ± 15.65 |
|  |  | Oral Isolated CBD 1.2 mg/kg every 12 h | 83.25 ± 29.57 | 79.5 ± 40.79 |  |  |  |
|  |  | Oral liposomal CBD 0.5 mg/kg every 12 h | 128.6 ± 86.28 | 124.6 ± 90.25 |  |  |  |
|  | ALP (U/L), mean ± SD  (normal 17-111 U/L) | Oral Isolated CBD 0.5 mg/kg every 12 h | 86.4 ± 59.7 | 95.2 ± 67.8 | Placebo oil (fractionated coconut oil) | 76.5 ± 15.2 | 81.2 ± 23.5 |
|  |  | Oral Isolated CBD 1.2 mg/kg every 12 h | 129.9 ± 21.2 | 138.5 ± 25.4 |  |  |  |
|  |  | Oral liposomal CBD 0.5 mg/kg every 12 h | 106.25 ± 44.9 | 147.5 ± 46.4 |  |  |  |
|  | Creatinine (mg/dL), mean ± SD (normal 0.6-1.4 mg/dL) | Oral Isolated CBD 0.5 mg/kg every 12 h | 1.34 ± 0.38 | 1.34 ± 0.34 | Placebo oil (fractionated coconut oil) | 1.25 ± 0.19 | 1.28 ± 0.22 |
|  |  | Oral Isolated CBD 1.2 mg/kg every 12 h | 1.375 ± 0.35 | 1.5 ± 0.42 |  |  |  |
|  |  | Oral liposomal CBD 0.5 mg/kg every 12 h | 1.125 ± 0.17 | 1.175 ± 0.377 |  |  |  |
|  | BUN (mg/dL), mean ± SD (normal 10-32 mg/dL) | Oral Isolated CBD 0.5 mg/kg every 12 h | 18.6 ± 6.22 | 18.6 ± 4.39 | Placebo oil (fractionated coconut oil) | 13.0 ± 4.24 | 14.5 ± 4.1 |
|  |  | Oral Isolated CBD 1.2 mg/kg every 12 h | 21.25 ± 4.5 | 16.5 ± 0.7 |  |  |  |
|  |  | Oral liposomal CBD 0.5 mg/kg every 12 h | 15.20 ± 6.16 | 15.0 ± 8.32 |  |  |  |
| Gamble,  2018 | CBPI – PSS (0-40), mean ± SD | Oral full-spectrum CBD oil 2 mg/kg every 12 h plus standard of care (NSAIDs, fish oil, and/or glucosamine sulfate, chondroitin sulfate) | 21 ± 8 | 14 ± 8 | Placebo oil (olive oil with 10 PPT anise oil and 5 PPT peppermint oil) plus standard of care (NSAIDs, fish oil, and/or glucosamine sulfate, chondroitin sulfate) | 17 ± 7 | 19 ± 9 |
|  | CBPI – PIS (0-60), mean ± SD |  | 35 ± 15 | 26 ± 14 |  | 27 ± 15 | 31 ± 16 |
|  | Hudson (0-110), mean ± SD |  | 54 ± 13 | 67 ± 10 |  | 65 ± 14 | 60 ± 19 |
|  | Veterinary lameness (1-5),  median (IQR) |  | 3 (1-4) | 3 (1-4) |  | 3 (2-4) | 3 (1-4) |
|  | Veterinary pain (1-5), median (IQR) |  | 3 (3-4) | 3 (1-4) |  | 3 (2-4) | 3 (2-4) |
|  | Veterinary weight-bearing (1-5),  median (IQR) |  | 2 (1-3) | 2 (1-3) |  | 2 (1-3) | 2 (1-3) |
|  | ALT (U/L), mean ± SD (normal 20-98 U/L) |  | 93 ± 86 | 114 ± 119 |  | 90 ± 89 | 166 ± 284 |
|  | ALP (U/L), mean ± SD  (normal 17-111 U/L) |  | 160 ± 212 | 323 ± 407 |  | 186 ± 287 | 175 ± 248 |
|  | Creatinine (mg/dL), mean ± SD (normal 0.6-1.4 mg/dL) |  | 1.0 ± 0.3 | 1.0 ± 0.3 |  | 0.9 ± 0.3 | 1.0 ± 0.3 |
|  | BUN (mg/dL), mean ± SD (normal 10-32 mg/dL) |  | 20 ± 9 | 20 ± 6 |  | 19 ± 6 | 19 ± 6 |
| Mejia,  2021 | CBPI – PSS (0-40), mean ± SD | Oral full-spectrum CBD oil 2.5 mg/kg every 12 h | 17.64 ± 6.37 | 14.73 ± 7.08 | Placebo oil | 17.64 ± 6.37 | 14.86 ± 5.74 |
|  | CBPI – PIS (0-60), mean ± SD |  | 32.76 ± 11.80 | 26.71 ± 13.12 |  | 32.76 ± 11.80 | 24.81 ± 12.91 |
|  | LOAD (0-52), mean ± SD |  | 28.00 ± 6.88 | 24.91 ± 8.05 |  | 28.00 ± 6.88 | 25.05 ± 8.48 |
|  | Total activity count, % change from baseline |  | - | -3.56 |  | - | -9.96 |
|  | %Peak vertical force normalized by body weight, mean ± SD |  | 52.32 ± 16.95 | 53.86 ± 19.11 |  | 52.32 ± 16.95 | 53.63 ± 18.69 |
|  | %Body weight distribution, mean ± SD |  | 21.59 ± 3.81 | 22.17 ± 4.03 |  | 21.59 ± 3.81 | 22.07 ± 4.37 |
|  | Liver enzymes elevation, n/N (%) |  | - | 14/23 (61%) |  | - | 1/23 (4%) |
|  | Vomiting, n/N (%) |  | - | 2/24 (8%) |  | - | - |

Abbreviations: ALP – alkaline phosphatase; ALT – alanine transaminase; AST – aspartate transaminase; BUN – blood urea nitrogen; CBD – cannabidiol; CBPI – canine brief pain inventory; GGT – gamma glutamyl transferase; HCPI – Helsinki Chronic Pain Index; LOAD – Liverpool Osteoarthritis in Dogs; NSAID – non-steroidal anti-inflammatory drug; PIS – pain interference score; PSS – pain severity score; QoL – quality of life.
